# Supplementary material for: Hidden vulnerability of US Atlantic coast to sea-level rise due to vertical land motion
Source: Nat Commun. 2023 Apr 11;14:2038. doi: 10.1038/s41467-023-37853-7 (PMC10090057; doi:10.1038/s41467-023-37853-7)
Supplement: Supplementary file 1 — Supplementary information [file 41467_2023_37853_MOESM1_ESM.pdf]

Supplementary Materials for

Hidden Vulnerability of US Atlantic Coast to Sea-level Rise due to Vertical Land Motion

Leonard O. Ohenhen*, Manoochehr Shirzaei, Chandrakanta Ojha, Matthew L. Kirwan
*Corresponding author. Email: ohleonard@vt.edu

This PDF file includes:

Supplementary Figs. 1 to 11
Supplementary Tables 1 to 3

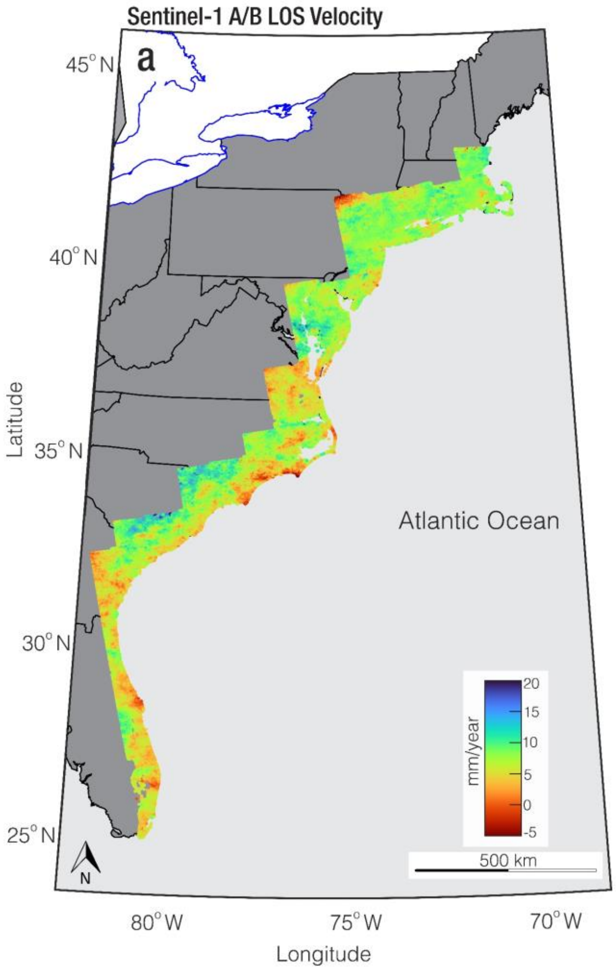

Fig. 1. Line of sight (LOS) deformation map. (a) LOS velocity obtained along the U.S. Atlantic Coast from Sentinel-1 C-band datasets in ascending viewing geometry during 2015 – 2020. (b) LOS velocity obtained along the U.S. Atlantic Coast from advanced land observing satellite (ALOS) L-band datasets in ascending viewing geometry during 2007 – 2011. Positive (negative) values denote movement towards (away from) the satellite. Supplementary table 1 summarizes the path and frame parameters of the SAR datasets. National, state, and great lakes boundaries in **(a)** and **(b)** are based on public domain vector map data by World DataBank (https://data.worldbank.org/) generated in MATLAB.

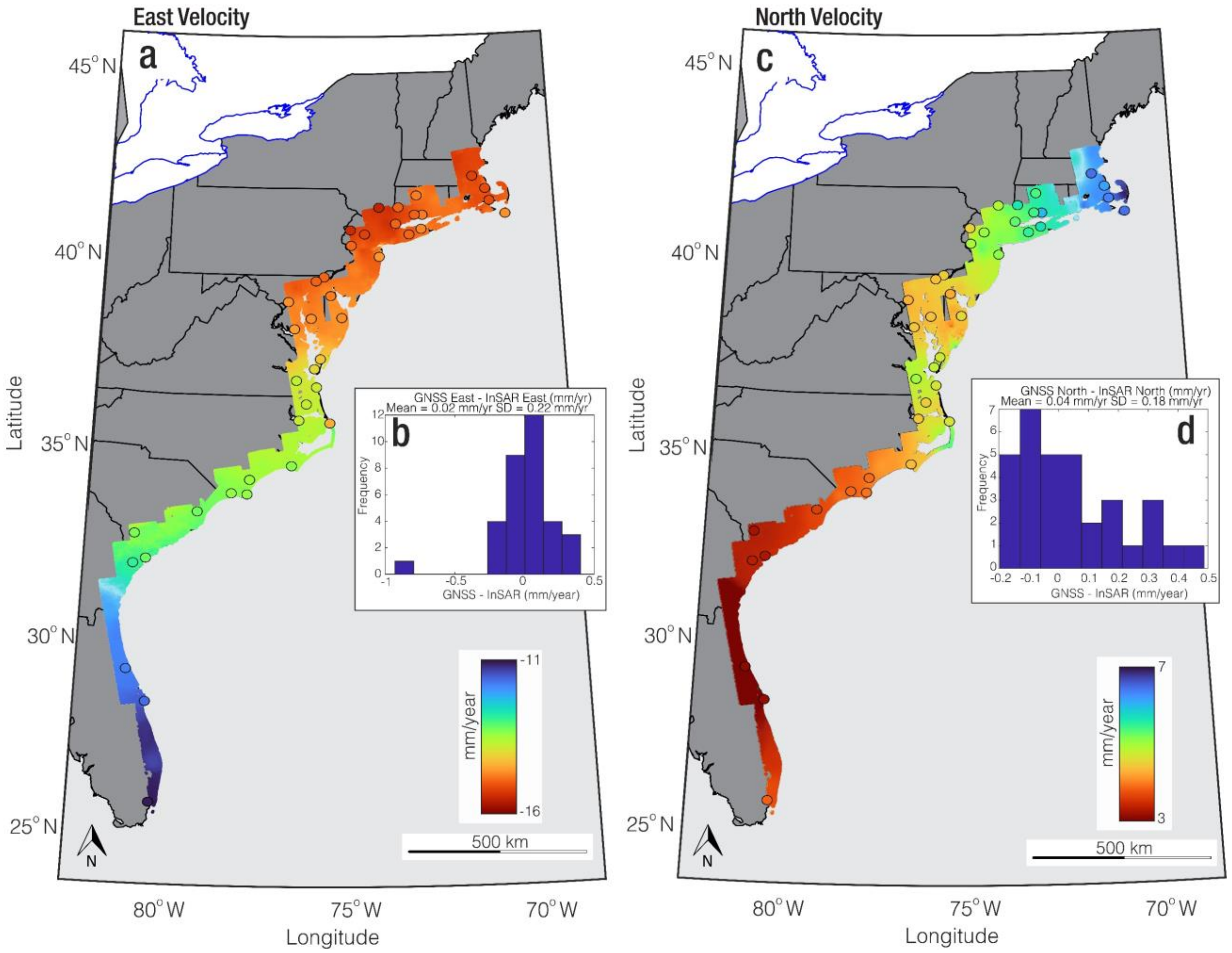

Fig. 2. Horizontal velocities across the US Atlantic coast. (a) Estimated east velocity. The circles show the location of GNSS validation observations color-coded with their respective east velocities. (b) Histogram comparing GNSS east rates with estimated east velocity. The standard deviation (SD) of the difference between the two datasets is 0.22 mm per year. (c) Estimated north velocity. The circles show the location of GNSS validation observations color-coded with their respective north velocities. (d) Histogram comparing GNSS north rates with estimated north velocity. The standard deviation (SD) of the difference between the two datasets is 0.18 mm per year. National, state, and great lakes boundaries in **(a)** and **(c)** are based on public domain vector map data by World DataBank (https://data.worldbank.org/) generated in MATLAB.

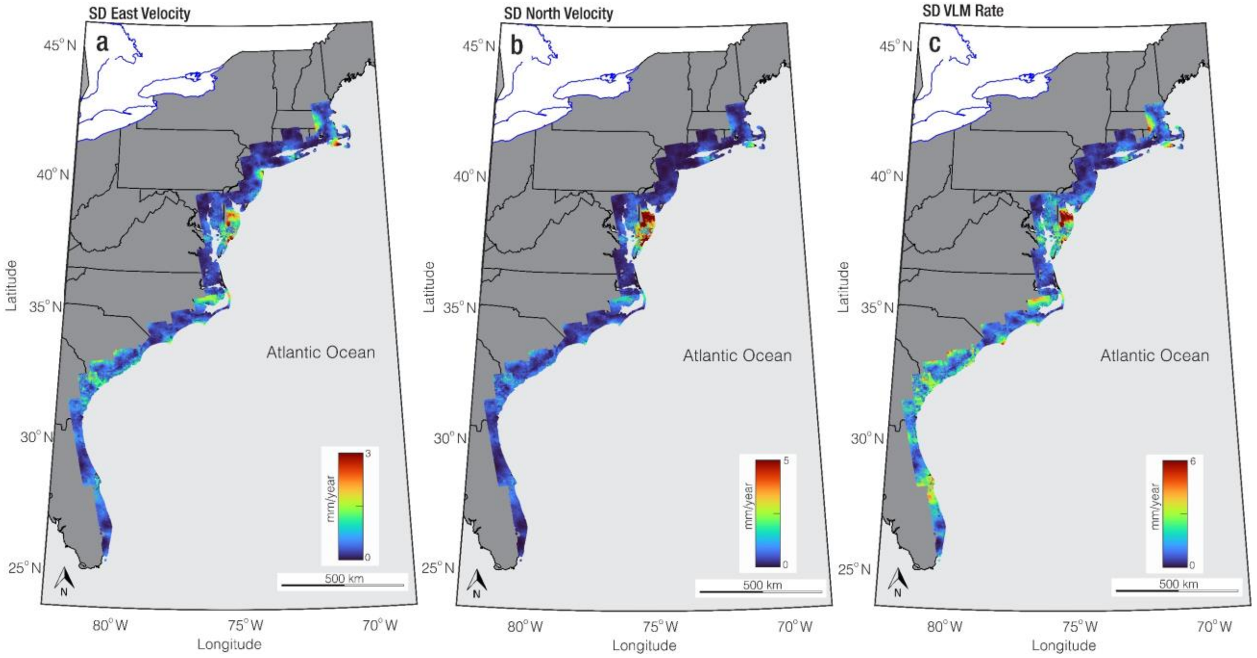

Fig. 3. 3D velocity standard deviation (SD) distribution maps. (a) east, (b) north, and (c) up. The units for the rate scale are mm per year. National, state, and great lakes boundaries in **(a), (b),** and **(c)** are based on public domain vector map data by World DataBank (https://data.worldbank.org/) generated in MATLAB.

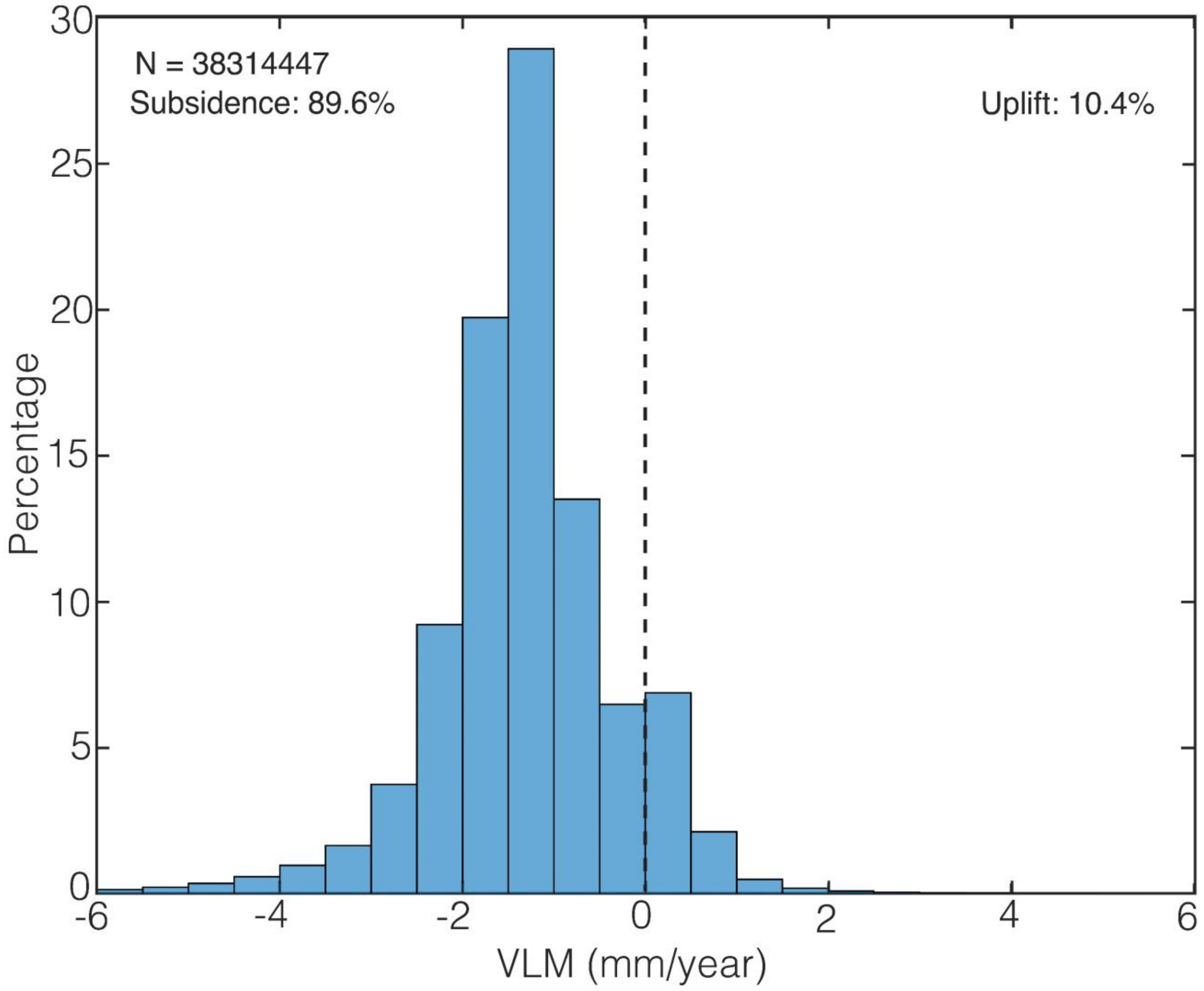

Fig. 4. Distribution of Vertical land motion (VLM). Histogram showing the distribution of VLM rate (mm per year). VLM rate < 0 indicates subsidence, while VLM rate ≥ 0 indicates uplift. N is the number of pixels.

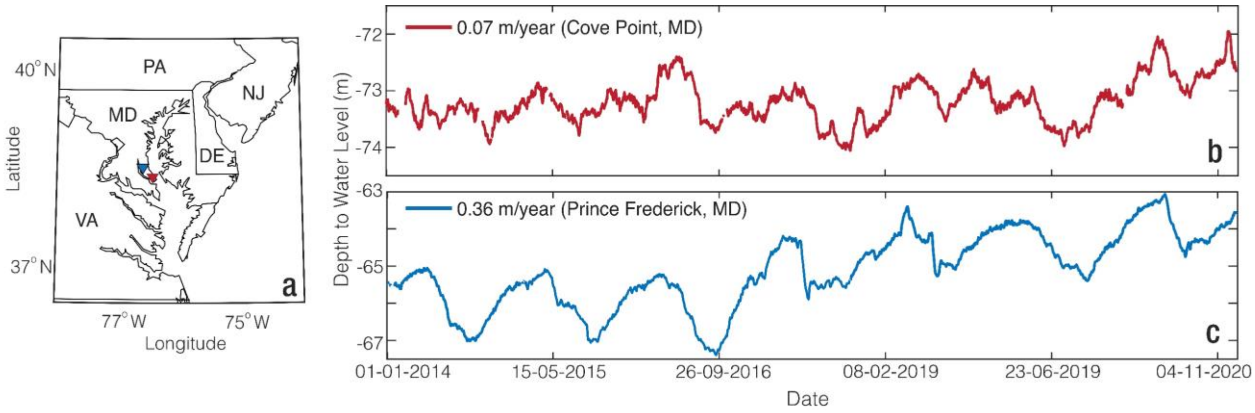

Fig. 5. Groundwater level during 2014 – 2020 in the Chesapeake Bay area. (a) Map of Chesapeake Bay area showing the well locations. The inverted triangle shows the location of the wells shown in B and C. The red triangle is well in Cove Point, Maryland (MD) shown in B, and the blue triangle is well in Prince Frederick Point, MD shown in C. (b) Depth to water level for well station at Cove Point, MD. The linear rate is 0.07 m per year. (c) Depth to water level for well station at Prince Frederick Point, MD. The linear rate is 0.36 m per year. The groundwater data is from the United States Geological Survey (USGS) groundwater data for the nation[55]. National and state boundaries in **(a)** are based on public domain vector map data by World DataBank (https://data.worldbank.org/) generated in MATLAB.

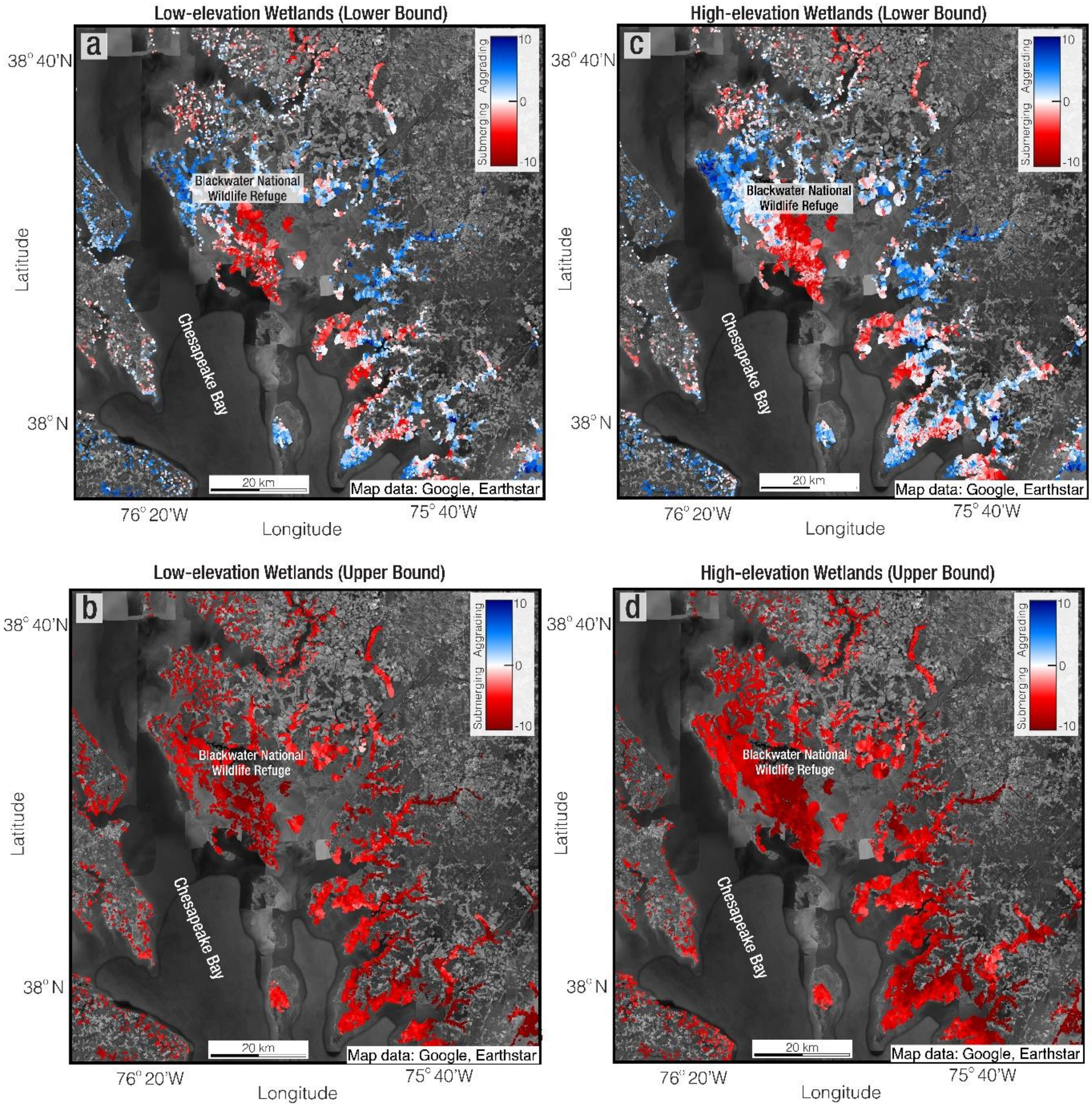

Fig. 6. Wetland vulnerability for the Chesapeake Bay area. (a) Lower bound of vertical resilience (VR) for low-elevation wetlands. (Background Image: Google, Earthstar). (b) Upper bound of VR for low-elevation wetlands. (Background Image: Google, Earthstar). (c) Lower bound of VR for high-elevation wetlands. (Background Image: Google, Earthstar). (d) Upper bound of VR for high-elevation wetlands. (Background Image: Google, Earthstar). VR is calculated as vertical land motion (VLM) minus sea level rise. The upper bounds and lower bounds are calculated as ± 2SD. Red colors indicate submerging wetlands (VR < -0.5 mm per year), blues indicate aggrading wetlands (VR > 0.5 mm per year), and whites indicate maintaining wetlands (-0.5 mm per year ≤ VR ≤ 0.5 mm per year).

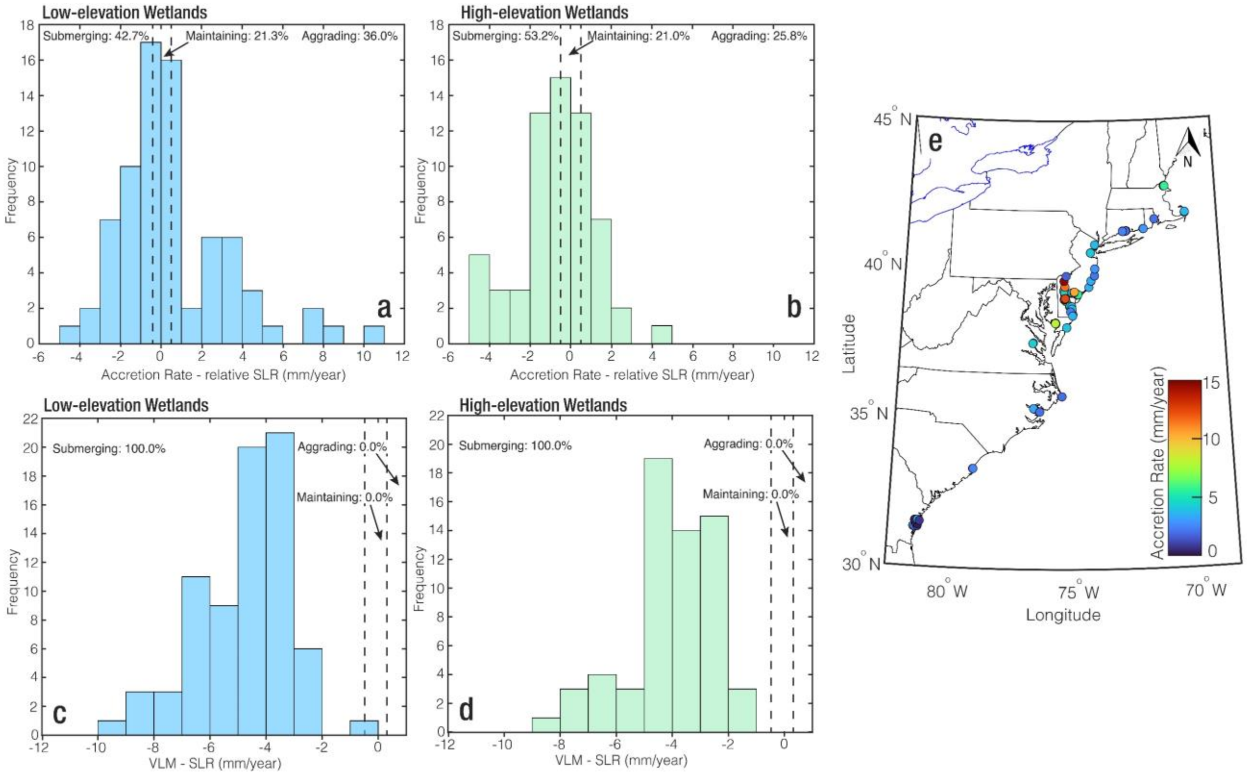

Fig. 7. Point estimates of wetland vulnerability across the US Atlantic coast. (a) Vertical resilience (VR) for low-elevation wetlands calculated as accretion rate minus relative sea-level rise (SLR). (b) VR for high-elevation wetlands calculated as accretion rate minus relative SLR. (c) VR for low-elevation wetlands calculated as vertical land motion (VLM) rate minus SLR. (d) VR for low-elevation wetlands calculated as VLM rate minus SLR. VR values less than -0.5 mm per year indicate submerging wetlands, VR values greater than 0.5 mm per year indicates aggrading wetlands, and VR values between -0.5 mm per year and 0.5 mm per year represent maintaining wetlands. (e) Accretion rate across the US Atlantic coast compiled from Kirwan *et al.*[38] and Holmquist *et al*.[64]. National, state, and great lakes boundaries in **(e)** are based on public domain vector map data by World DataBank (https://data.worldbank.org/) generated in MATLAB.

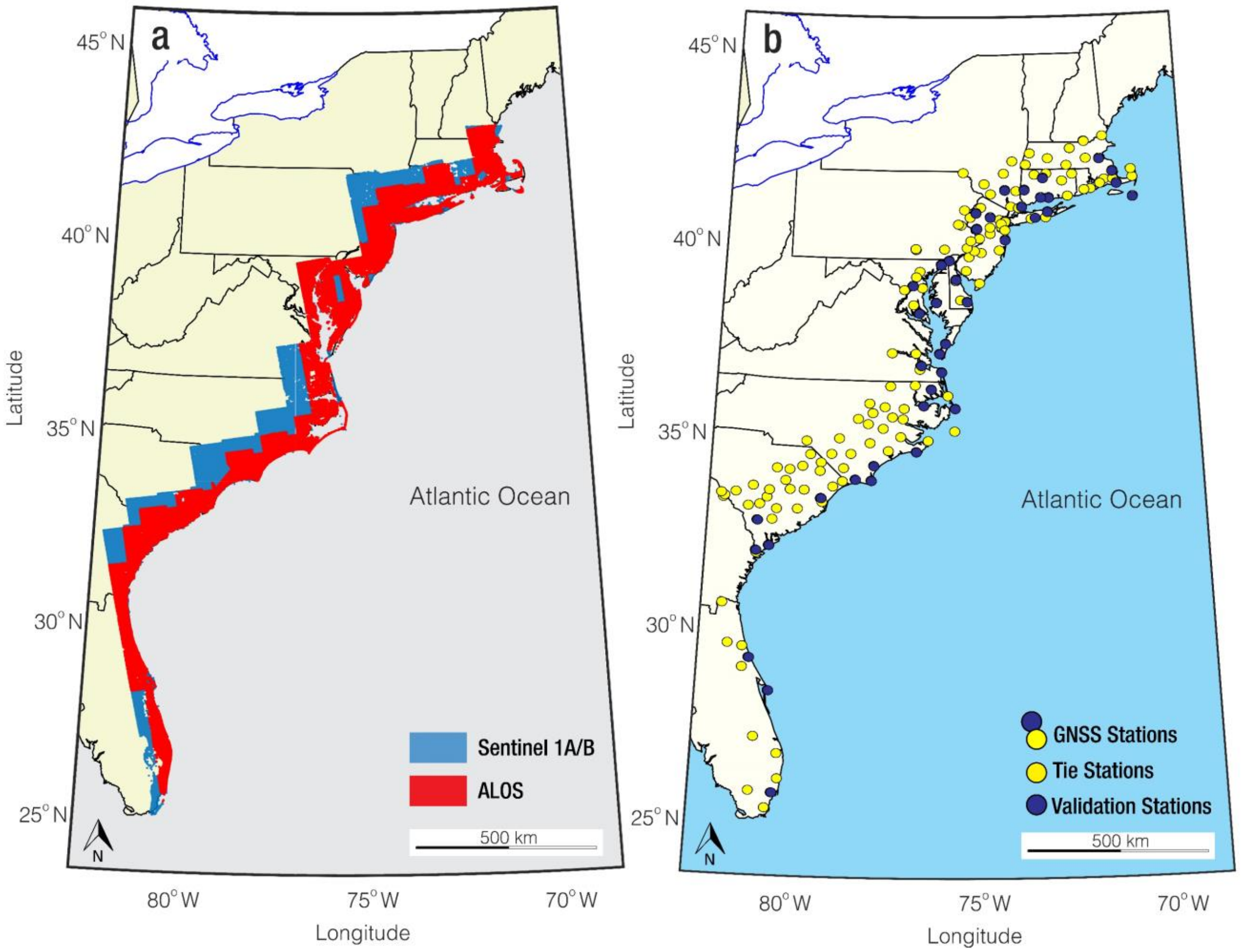

Fig. 8. Synthetic Aperture Radar (SAR) and global navigation satellite system (GNSS) datasets. (a) The final location of SAR pixels after transformation to a global reference frame. The blue colors indicate the locations of Sentinel-1 pixels. The red colors indicate the locations of ALOS pixels. (b) Locations of GNSS stations used in the study. The yellow circles are the locations of 132 GNSS tie stations used in the study. The blue circles are the locations of 41 GNSS stations used in validation. National, state, and great lakes boundaries in **(a)** and **(b)** are based on public domain vector map data by World DataBank (https://data.worldbank.org/) generated in MATLAB.

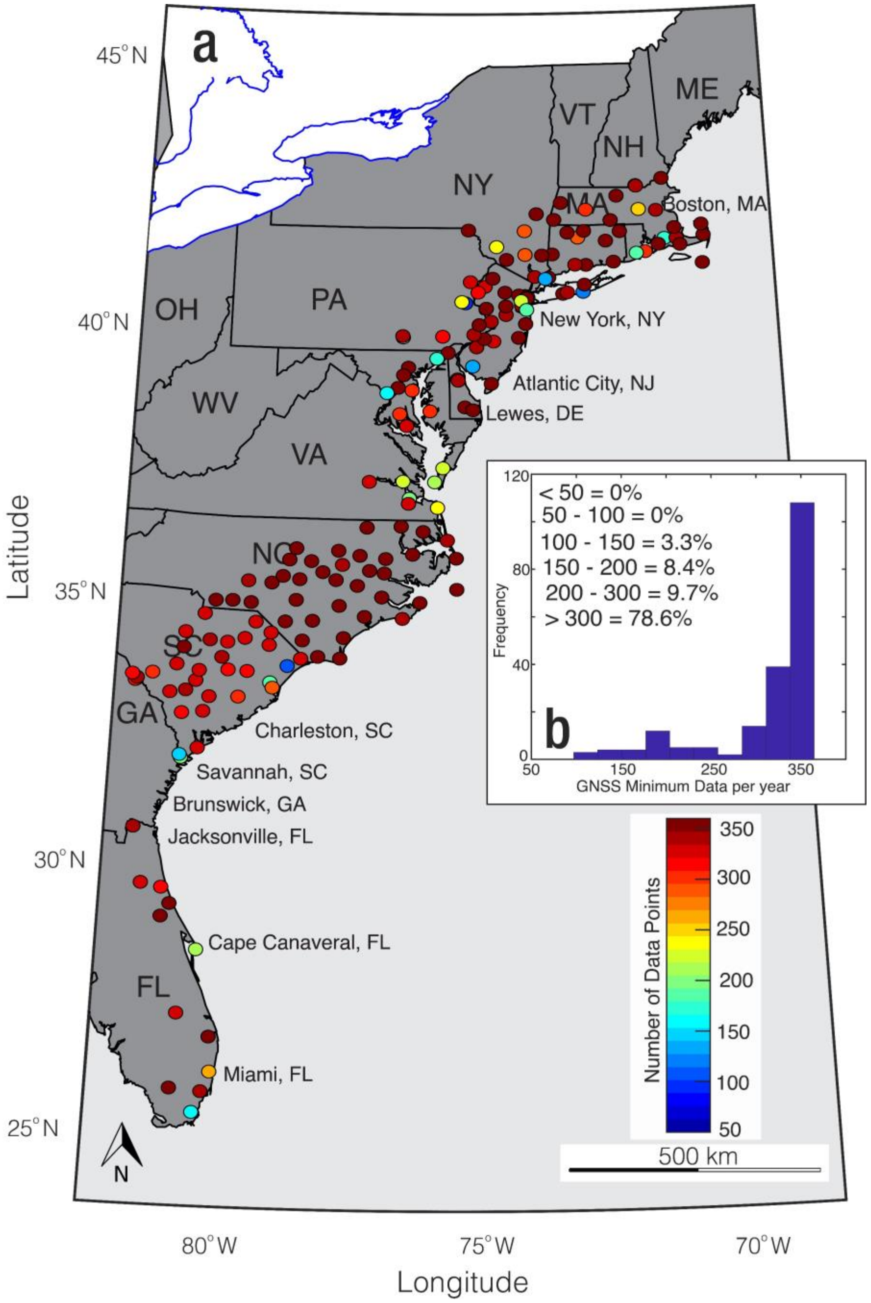

Fig. 9. Minimum yearly global navigation satellite system (GNSS) samples. (a) Distribution of the minimum number of samples per year for each GNSS station. (b) Histogram showing the distribution of minimum yearly samples for each GNSS station. National, state, and great lakes boundaries in **(a)** are based on public domain vector map data by World DataBank (https://data.worldbank.org/) generated in MATLAB.

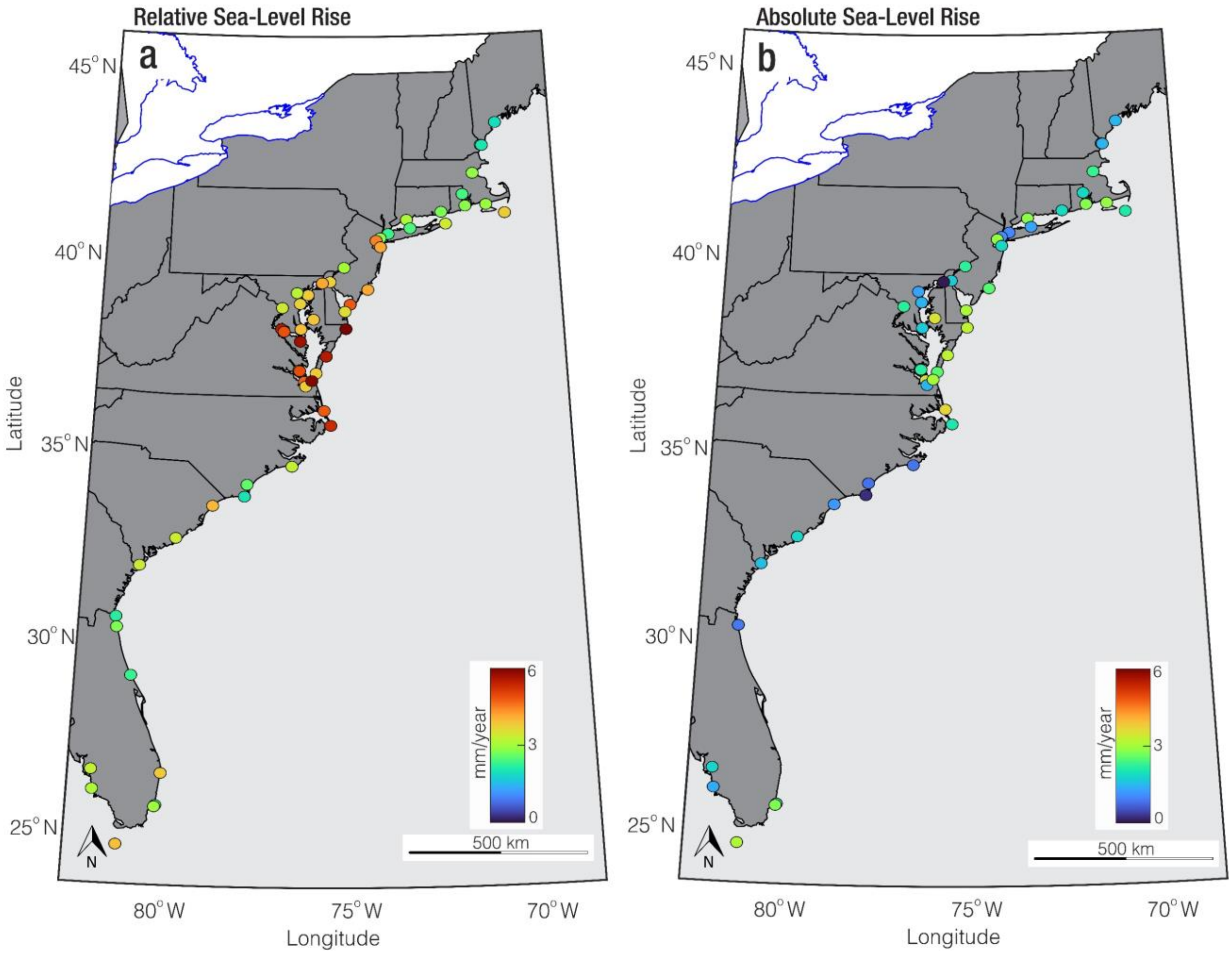

Fig. 10. Sea-level rise (SLR) from tide gauges across the US Atlantic coast. (a) Relative SLR[99]. (b) Absolute SLR corrected for Vertical land motion using the median up velocity of GNSS stations. National, state, and great lakes boundaries in **(a)** and **(b)** are based on public domain vector map data by World DataBank (https://data.worldbank.org/) generated in MATLAB.

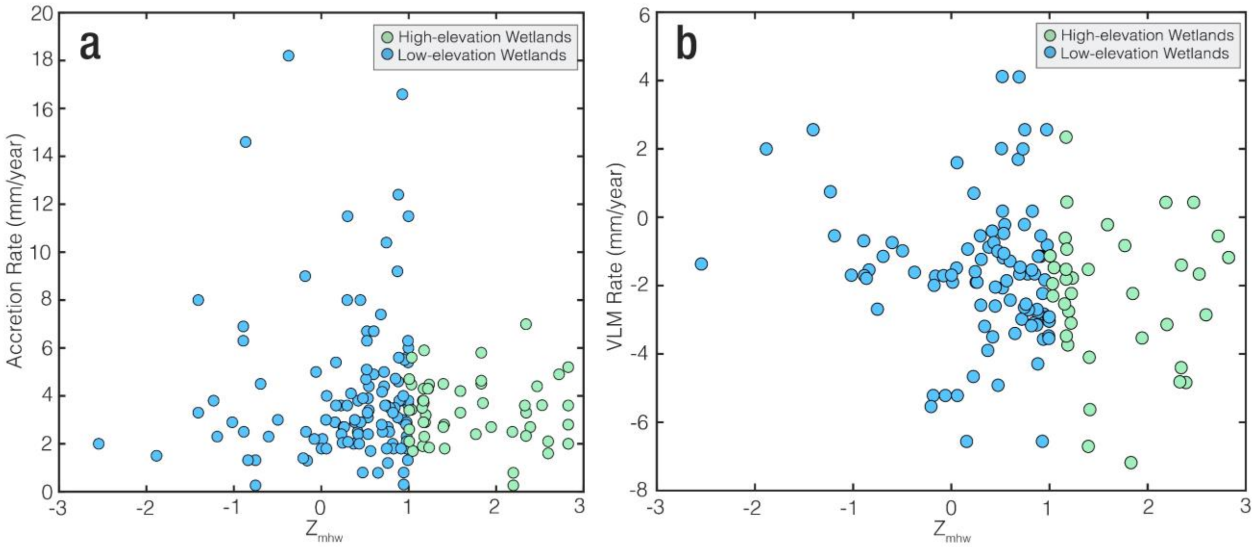

Fig. 11. Elevation normalized to mean high water (Z_{MHW}) for point accretion data across the US Atlantic coast. (a) Plot of accretion rate in mm per year versus Z_{mhw} for point accretion data shown in Supplementary Fig. 7e. (b) Plot of vertical land motion (VLM) rate in mm per year versus Z_{mhw}. VLM rates were extracted for each point with measurements of accretion rate. The blue symbols are low-elevation wetlands with Z_{mhw} less than 1, and the green symbols are high-elevation wetlands with Z_{mhw} greater than or equal to 1.

Table 1. Synthetic Aperture Radar datasets used in the analysis.

Sentinel-1A/B			ALOS			ALOS		
**Orbit**	**Path**	**Frames**	**Orbit**	**Path**	**Frames**	**Orbit**	**Path**	**Frames**
Ascending	48	79	Ascending	116	880, 890	Ascending	133	770, 780
Ascending	48	84	Ascending	117	880, 890	Ascending	134	750 - 780
Ascending	48	89	Ascending	118	880	Ascending	135	740, 750, 780
Ascending	48	94	Ascending	119	870, 880	Ascending	136	690 - 780
Ascending	48	99	Ascending	120	870, 880	Ascending	137	690 - 770
Ascending	150	102	Ascending	121	870	Ascending	138	680, 690
Ascending	77	107	Ascending	122	860 - 870	Ascending	139	680
Ascending	4	110	Ascending	123	820, 830, 860	Ascending	140	680
Ascending	4	115	Ascending	124	820 - 860	Ascending	141	670
Ascending	106	118	Ascending	125	820 - 840	Ascending	142	660, 670
Ascending	106	123	Ascending	126	820	Ascending	143	660
Ascending	33	125	Ascending	127	810, 820	Ascending	144	650
Ascending	33	130	Ascending	128	810, 820	Ascending	145	640, 650
Ascending	135	131	Ascending	129	800, 810	Ascending	146	640
Ascending	62	133	Ascending	130	800, 810	Ascending	147	630, 640
Ascending	62	138	Ascending	131	800, 810	Ascending	148	500 - 560, 610 - 630
Ascending	164	142	Ascending	132	780 - 800	Ascending	149	560 - 610

Table 2. Comparison of IPCC vertical land motion (VLM) rate[56,57] (mm per year) with InSAR VLM rate from this study (mm per year). Note that a negative VLM (or subsidence) in a positive sea level change. The percent (%) difference is calculated as: $\frac{IPCC\ VLM - InSAR\ VLM}{(IPCC\ VLM + InSAR\ VLM/2)} \times 100\%$. A negative % difference indicates underestimation and a positive difference indicates overestimation, with a ±20% error buffer.

Tide Gauge Station	IPCC VLM Rate (mm per year)	VLM Rate from this Study (mm per year)	% Difference
Boston, MA	-1.1±0.2	-1.0±0.1	9.5
Woods Hole, MA	-1.4±0.2	-2.0±0.1	-35.3
Bridgeport, CT	-1.2±0.2	-1.2±0.1	0.0
Atlantic City, NJ	-2.4±0.2	-3.1±0.2	-25.5
Cape May, NJ	-2.0±0.2	-3.5±0.3	-54.5
Lewes, DE	-2.3±0.2	-2.7±0.7	-16.0
Sewells Point, VA	-2.9±0.2	-2.2	27.5
Beaufort, NC	-1.6±0.4	-2.2±0.2	-31.6
Wilmington, NC	-1.0±0.3	-1.6±0.3	-46.2
Charleston I, SC	-1.7±0.2	-3.0±0.5	-55.3
Trident Pier, FL	-0.8±0.4	-1.8±0.1	-76.9
Miami Beach, FL	-1.1±0.4	-0.9±0.4	20.0

Table 3. Comparison of SET-MH elevation change rate[45] (mm per year) with InSAR vertical land motion (VLM) rate (mm per year).

Longitude	Latitude	Elevation Change Rate (mm per year)	InSAR VLM (mm per year)
-81.2183	29.6290	-0.133	-0.163
-81.2880	29.7715	1.461	0.822
